# Assessment of the effectiveness of exercise interventions in the treatment of PTSD: based on a systematic evaluation and meta-analysis

**DOI:** 10.3389/fpsyg.2025.1702199

**Published:** 2026-01-07

**Authors:** Yifan Fang, Qilin Zhang, Zhiyi Lin

**Affiliations:** 1 School of Physical Education and Health Science, Guangxi Minzu University, Nanning, China; 2 School of Physical Education, Hunan Normal University, Changsha, China; 3 School of Physical Education and Sport Science, Fujian Normal University, Fuzhou, China

**Keywords:** exercise interventions, meta-analysis, post-traumatic stress disorder, PTSD, systematic evaluation

## Abstract

**Background:**

Physical exercise is increasingly recognized for its potential to alleviate symptoms of post-traumatic stress disorder (PTSD). However, uncertainty remains regarding which exercise modalities are most effective, optimal prescription parameters, and which patient populations benefit most. This study systematically evaluated the effects of various exercise interventions on PTSD symptoms, aiming to provide an evidence base for personalized treatment strategies.

**Methods:**

We conducted a systematic search of PubMed, APA PsycINFO, Embase, Cochrane Library, and Web of Science for randomized controlled trials (RCTs) examining exercise interventions in patients with PTSD. The search was completed in November 2024, and 14 RCTs meeting eligibility criteria were included. The study followed PRISMA guidelines and was registered in PROSPERO (CRD420250652205). A random-effects model was used for meta-analysis, with subgroup analyses performed to explore heterogeneity.

**Results:**

The meta-analysis showed that exercise interventions significantly reduced PTSD symptoms compared to control conditions (SMD = −0.35, 95% CI: −0.56 to −0.15, *p* < 0.05). Subgroup analyses indicated that yoga (SMD = −0.56, 95% CI: −0.85 to −0.27, *p* < 0.001) and resistance training (SMD = −0.38, 95% CI: −0.72 to −0.03, *p* = 0.031) were particularly effective. The most beneficial intervention protocol consisted of sessions delivered three times per week for 30–60 min, over a 12-week period. Additionally, female patients appeared to derive greater benefit from exercise interventions (SMD = −0.41, 95% CI: −0.76 to −0.06, *p* = 0.020).

**Conclusion:**

This review supports the use of structured exercise, especially yoga and resistance training, as an effective adjunctive intervention for PTSD. The findings inform evidence-based recommendations for clinical practice, including optimal dosing and population-specific considerations. Future research should integrate body-oriented frameworks such as psychomotor therapy to further elucidate mechanisms and personalize exercise-based approaches in trauma recovery.

## 1 Introduction

There’s a notable rise in the occurrence of PTSD and related mental health issues amid ongoing global conflicts and repeated natural calamities (Charlson et al., 2019). Notably, individuals directly or indirectly exposed to traumatic events including war, natural disasters, and catastrophic incidents face a heightened risk of PTSD development (Qi et al., 2016; Watkins et al., 2018). The effects of PTSD are multifaceted and significant. According to the DSM-5, PTSD is defined by four main symptom groupings: (1) intrusive recollections of the traumatic event, often experienced as nightmares or distressing memories; (2) persistent avoidance of reminders linked to trauma; (3) ongoing negative shifts in emotional and psychological conditions; and (4) heightened alertness, which includes symptoms like irritability, insomnia, and heightened alertness. These symptoms can severely impact psychosocial functioning and are associated with other health issues, such as obesity, depression, chronic pain, substance abuse disorders, and suicidal ideation (Hegberg et al., 2019). Furthermore, PTSD is comorbid with numerous physical conditions, including metabolic syndrome, compounding the overall health burden on affected individuals. Consequently, early identification, intervention, and treatment of PTSD are critical to alleviating the societal and individual burdens imposed by this disorder (Warshaw et al., 1993; Zatzick et al., 2002). Presently, trauma-focused psychotherapy is the cornerstone of first-line treatment for PTSD, as delineated in international guidelines such as those from the International Society for Traumatic Stress Studies (ISTSS) (Martin et al., 2021). Among the recommended modalities, Prolonged Exposure (PE) is a well-established approach, typically conducted over 8–12 weeks, where therapists guide patients to gradually process trauma-related emotions through imaginal and *in vivo* exposure (Foa et al., 2007). Additionally, guidelines strongly endorse other evidence-based therapies, including Cognitive Processing Therapy (CPT), which focuses on identifying and challenging trauma-related maladaptive beliefs, and Eye Movement Desensitization and Reprocessing (EMDR), which facilitates adaptive memory processing through bilateral sensory stimulation (Martin et al., 2021).

Although these gold-standard psychotherapies have proven effective for many individuals, resulting in a significant proportion no longer meeting PTSD criteria post-treatment (Resick et al., 2012), they are not universally effective. Significant challenges persist in the treatment of PTSD, including high dropout rates often attributed to the emotional distress associated with confronting traumatic memories, limited efficacy observed in specific populations such as combat veterans (Straud et al., 2019), and the scarcity of access to adequately trained mental health providers. These limitations underscore the critical need to develop and evaluate alternative or adjunctive intervention strategies. In this context, physical exercise has emerged as a promising intervention, offering cost-effectiveness and low risk, with the potential to alleviate PTSD symptoms through various physiological mechanisms, such as regulating the hypothalamic–pituitary–adrenal (HPA) axis, and psychological pathways like enhancing self-efficacy and reducing intrusive thoughts (Garber et al., 2011; Hegberg et al., 2019).

A growing body of evidence supports physical exercise as a scientifically validated and cost-effective intervention for PTSD, either as a standalone treatment or, more commonly, as an adjunct to first-line psychotherapies (e.g., prolonged exposure) or pharmacotherapy (Garber et al., 2011; Hegberg et al., 2019). These interventions, frequently delivered in group settings to foster social support, encompass a diverse range of modalities. These include mind–body exercises (e.g., yoga, tai chi) which integrate physical movement with breath control and mindfulness, resistance training, aerobic workouts (e.g., running, cycling), and mixed or integrated programs that combine these elements. The therapeutic benefits of exercise for PTSD are thought to stem from multiple physiological and psychological mechanisms. Physiologically, exercise may help regulate the dysregulated stress response systems in PTSD, including the hypothalamic–pituitary–adrenal (HPA) axis and the autonomic nervous system, thereby reducing hyperarousal and improving allostatic load (Streeter et al., 2012). Psychologically, it can promote a sense of self-efficacy, provide a constructive distraction from intrusive thoughts, and facilitate a non-threatening reconnection with bodily sensations. Previous meta-analyses have generally demonstrated that these exercise interventions lead to a significant, albeit often small to moderate, reduction in PTSD symptoms compared to non-exercise control groups (e.g., waitlist, treatment-as-usual, or health education) (Oppizzi and Umberger, 2018; Rosenbaum et al., 2015a; Whitworth and Ciccolo, 2016). For instance, specific studies and reviews have highlighted the efficacy of mind–body practices like yoga and tai chi (Tan et al., 2023; Laplaud et al., 2023), as well as resistance training (Whitworth et al., 2019a; Whitworth et al., 2019b). However, the evidence is not entirely consistent, with some studies indicating that certain exercise modalities may not be superior to other active interventions (Young-McCaughan et al., 2022) or may show limited effects for specific subpopulations (Kysar-Moon et al., 2021). This inconsistency underscores the need to critically evaluate the strengths, weaknesses, and applicability of different exercise interventions to optimize PTSD treatment strategies. Although yoga is regarded as an adjunctive treatment of PTSD in many systematic reviews (Rosenbaum et al., 2015b; Zhu et al., 2022), there are still many disagreements and gaps in this field, and updated and general work is needed to clarify these issues. For instance, early studies (Rosenbaum et al., 2015a) provided the first proof for this field, and later studies (Zhu et al., 2022; Yuan et al., 2025) generally showed positive effects of physical exercise on core PTSD symptoms and related disorders. However, as evidence builds, important issues emerge: Zhu et al., 2022) showed that yoga does not have better results than control groups in treating depression (Martinez-Calderon et al., 2024). Finally, the relative effectiveness of different exercise modalities (yoga, resistance training, and multimodal exercise) and optimal exercise prescriptions for different populations (e.g., gender differences) is not clear.

To move from a comparative analysis of exercise to a mechanism-based understanding, we recommend existing clinical frameworks focusing on the mind–body connection (Kim et al., 2013). Psychomotor therapy is a body-based therapy based on movement, bodily awareness, and emotion processing, often used in clinical settings to help people with PTSD regulate their moods, regain bodily safety, and process trauma nonverbally (Koch et al., 2019). Through distinct theoretical approaches and techniques, psychomotor therapy complements and enriches the general evidence supporting embodied interventions for trauma recovery (van Westrhenen and Fritz, 2014).

Therefore, by integrating insights from such body-oriented frameworks, this study aims to resolve contradictions in current research through an updated systematic review and meta-analysis, providing more actionable personalized solutions for clinical practice. Specifically, the incremental contributions of this study are: (1) Focus on controversy analysis: Prioritizing comparisons between different exercise modalities like yoga and resistance training to uncover new insights for resolving aforementioned contradictions; (2) Deepening prescription exploration: Not only examining overall exercise effects but also conducting detailed subgroup analyses (e.g., intervention duration, frequency, gender subgroups) to explore moderating factors of efficacy; (3) Methodological enhancement: Employing rigorous criteria to screen randomized controlled trials and conducting comprehensive assessments of primary outcome measures to generate more reliable, consolidated evidence. In summary, this study moves beyond a mere synthesis of existing evidence, aiming to bridge a persistent knowledge gap. By clarifying the most effective exercise modalities and parameters, we seek to establish an empirical foundation that can inform the future design of structured, body-oriented interventions, including trauma-focused psychomotor therapy protocols.

## 2 Methods

### 2.1 Protocol for reporting and registration

The International Registry has documented this study for systematic evaluation under Prospective Systematic Evaluation (PROSPERO) with registration number CRD420250652205. In the execution of the meta-analysis and systematic review, strict compliance was maintained with the PRISMA 2020 guidelines, which are highly preferred for systematic reviews and meta-analyses.

### 2.2 Databases and search strategy

As of November 2024, five literature databases (PubMed, APA PsycINFO, Embase, Cochrane Library, Web of Science) were searched. Utilizing EndNote (version X9) reference management software, every article retrieved from each database underwent sorting and duplication verification. The efficacy of exercise interventions for PTSD was assessed for published randomized controlled trials (RCTs) of searches. PTSD and associated psychopathology in patients with PTSD were examined. Although systematic reviews and meta-analyses themselves were excluded, their reference lists were screened to identify any additional eligible primary studies that may have been missed by the electronic database search. Search terms included ‘stress disorder’, ‘post-traumatic’, and ‘exercise’, along with their respective Medical Subject Headings (MeSH), encompassing terms, keywords, and synonyms. A pair of investigators (first authors) independently executed the searches, and in case of any inconsistencies, a third investigator (corresponding author) reviewed the varied findings prior to finalizing a decision. If any discrepancies occurred, the different results were reviewed by a third researcher (corresponding author) to make a final decision.

### 2.3 Criteria for qualifying

Criteria for inclusion were set based on the PICOS methodology, covering aspects like Study Population, Intervention, Comparison, Study Design, and Outcome. Studies qualified for inclusion based on the following criteria: (1) Study Population: Individuals aged 18 years and older diagnosed with PTSD, including active-duty military personnel, refugees, veterans, and other diverse groups. The study aimed to encompass a broad demographic to facilitate a comprehensive meta-analysis of activity intensity. (2) Intervention: Physical activity interventions, such as yoga, resistance training, mixed exercise, aerobic exercise, and other modalities. No specific limitations were applied to the dose, frequency, duration, or quality of the interventions. (3) Comparison: Control groups received various interventions, including time-matched attention, health education, social regulation, and monitoring. (4) Outcome: PTSD symptom severity assessed using standardized scales (e.g., PCL-5 or CAPS, with CAPS scores≥45) or comparable measures. (5) Study Design: Randomized controlled trials (RCTs) published in peer-reviewed journals.

### 2.4 Exclusion criteria

(1) Assessments, publications, editorial remarks, case studies, summaries of conferences, non-published papers, and Writings not composed in English were omitted.
(2) Research missing quantitative data or related outcome metrics was omitted.
(3) Articles that could not be accessed via various sources and techniques were omitted.
(4) Studies that lacked a control or comparison group, or where the control group had a high rate of missing data (as defined in our quality assessment criteria) that was not adequately addressed.
(5) Studies of non-exercise interventions like breathing, meditation, orthomolecular therapy, were excluded.

### 2.5 Study selection and data extraction

Every article obtained was transferred into EndNote X9 software for subsequent deduplication. A pair of primary authors (first authors) undertook an extensive literature survey, scrutinizing titles, abstracts, and entire texts. When there was a dispute over adding an article, the ultimate verdict was made after a period of consultation. With the assistance of a third researcher, the corresponding author. Information gathered from these studies included broad details (authors, year of publication, country of origin, gender, and age), essential attributes (size of the sample, type of intervention, length of intervention, weeks of intervention), and measures of outcomes. The main focus of this analysis was on PTSD symptoms, with secondary results encompassing anxiety, depression, and the quality of sleep. Independently, a pair of writers will distill and condense the result data, encompassing the average and variability of the unprocessed data. Conflicts will be settled via dialogue until agreement is reached or through discussions with the pertinent writer. For articles whose full text was not readily accessible through database subscriptions, we utilized our institutional library’s inter-library loan service to secure a copy. Furthermore, if the full text was retrieved but essential quantitative data (e.g., means, standard deviations) were missing for meta-analysis, we made attempts to contact the corresponding authors of the studies via email to request the required data. A follow-up email was sent after 2 weeks if no initial response was received.

### 2.6 Quality assessment

Two primary researchers (first authors) independently evaluated the risk of bias for the included randomised controlled trials using the Cochrane Risk of Bias tool for Randomized Trials, version 2 (ROB 2.0) (Sterne et al., 2019). The tool assesses bias across five domains: (1) bias arising from the randomization process, (2) bias due to deviations from intended interventions, (3) bias due to missing outcome data, (4) bias in measurement of the outcome, and (5) bias in selection of the reported result.

Judgments for each domain were made based on the following pre-defined criteria:

Randomization process: Studies were rated “low risk” if they described a random component in the sequence generation process (e.g., computer-generated random numbers) and adequate allocation concealment (e.g., central allocation; sequentially numbered, opaque, sealed envelopes). If methods were inappropriate or not described, the risk was rated “high” or “some concerns.”

Deviations from intended interventions: We assessed the effect of assignment to the intervention (intention-to-treat effect). Studies were rated “low risk” if participants and personnel were blinded, or if blinding was broken but the analysis was appropriately performed. Lack of blinding and inappropriate analysis led to a “high risk” rating.

Missing outcome data: Studies were rated ‘low risk’ if the proportion of missing outcomes was insufficient to materially affect the outcome (we pre-specified a threshold of <20% attrition), and if the reasons for missingness were balanced across groups and unlikely related to the true outcome. If high attrition (>20%) occurred and was not adequately addressed using appropriate methods (e.g., intention-to-treat analysis, multiple imputation), the risk was rated “high.”

Measurement of the outcome: Studies were rated “low risk” if the outcome assessors were blinded to the intervention status, given that PTSD scales, even if self-reported, can be influenced by knowledge of the intervention.

Selection of the reported result: Studies were rated “low risk” if the published report included all pre-specified outcomes, or if an analysis plan was registered and followed.

The overall risk of bias for each study was then judged as “low,” “some concerns,” or “high” according to the ROB 2.0 algorithm. Any disagreements between the two reviewers were resolved through discussion or by consultation with the corresponding author.

### 2.7 Data analysis

Statistical analysis of the incorporated studies was conducted using the Stata14 and 16 software. The effect size for each study was calculated as the standardized mean difference (SMD) between the exercise intervention group and the control group in the post-intervention PTSD symptom scores. The SMD, specifically Cohen’s d, was computed using the following formula:


d=(Me−Mc)/SDpooled

where Me and Mc are the post-intervention means of the exercise and control groups, respectively, and SD pooled is the pooled standard deviation of both groups. The calculated standardized mean difference (SMD) directly reflects the comparative effect of the exercise intervention relative to the control group beyond the observed changes. Therefore, the reported significant standardized mean difference indicates that the exercise intervention resulted in a greater reduction in post-traumatic stress disorder symptoms compared to the control group. A *p*-value less than 0.05 indicates a statistically significant variance. The diversity in outcomes across the studies was evaluated and measured through the I^2^ tool. A random-effects model was applied to the meta-analysis when I^2^ fell below 50%; otherwise, random effects were employed. To assess the differences in the total effect size, sensitivity analyses were conducted, entailing the exclusion of each study separately. To investigate the roots of this diversity, meta-regression was utilized. Moreover, in cases of asymmetry, the cut method was employed to detect and rectify any asymmetry in the funnel plot caused by publication bias. In addition to statistical significance, we evaluated the clinical significance (or clinical meaningfulness) of the findings. For the primary outcome of PTSD symptoms, a standardized mean difference (SMD) of 0.2, 0.5, and 0.8 are conventionally considered to represent small, medium, and large effect sizes, respectively (Leppink et al., 2016). Based on established guidelines in psychological trauma research, an SMD of ≥ 0.5 (a medium effect size) was predefined as indicative of a clinically meaningful change in PTSD symptoms (Hedges and Olkin, 1985). This threshold aligns with empirical data on what constitutes a meaningful difference from both patient and clinician perspectives.

## 3 Results

### 3.1 Literature search

**Figure 1:**
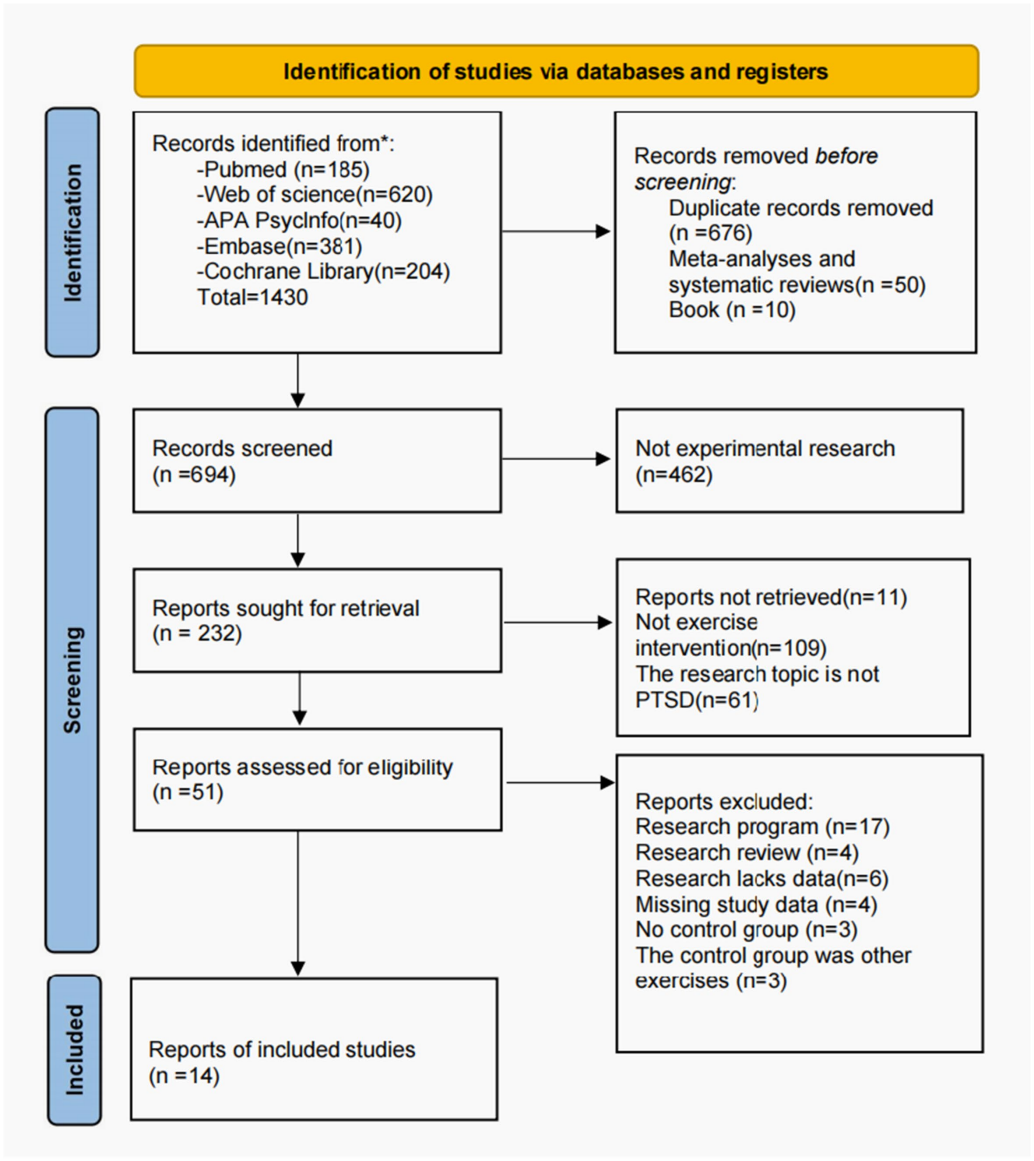
PRISMA flowchart. Systematic evaluation and meta-analysis flowchart for inclusion and exclusion.

Based on the predefined search strategy, an initial retrieval of 1,430 relevant publications was conducted across five databases. Following the exclusion of 676 duplicate records, 10 monographs, and 50 meta-analyses and systematic reviews, the remaining literature underwent title and abstract screening, resulting in the removal of 462 non-experimental studies. Subsequently, the full texts of the remaining 232 papers were downloaded and subjected to detailed review. Ultimately, 11 studies and a further 109 papers that did not involve exercise interventions or where the research topic was not PTSD were excluded. A further 37 papers that failed to meet the criteria were then removed, resulting in the final inclusion of 14 studies that met the criteria. The literature screening process is illustrated in Figure 1.

### 3.2 Study characteristics

#### 3.2.1 Participant characteristics

This analysis encompassed 14 publications released between 2014 and 2024. The majority of the relevant studies were conducted in five countries: the USA, Australia, Denmark, Germany, and Canada. All included studies were randomized controlled trials that focused on the impact of exercise therapies on PTSD management. The research involved 1,119 participants aged 18 to 68 years, with 544 in the exercise intervention group and 575 in the control group. Sample sizes varied from 15 to 323. Table 1 outlines the core attributes of the papers mentioned.

**Table 1:** Presents a comprehensive review and meta-analysis of various studies in the literature regarding the effectiveness of exercise therapy in treating post-traumatic stress disorder.

Study	n	Male/female	Included in analysis	Population	Age	Diagnostic criteria	Control	Interventions	Intervention characteristics	Intervention time	PTSD outcome measure
Bryant et al. (2023) Australia	130	79/51	130	Adults 18 years of age or older with a clinical diagnosis of PTSD	39.1 ± 14.4	5-point scale based on each of the past month’s symptoms (range 0–136; higher scores indicate greater PTSD severity)	Passive Stretch Group	Aerobics Group Personal aerobic target heart rate, i.e., 65–85% of maximum heart rate 9 times per week	Adjunctive Exposure therapy	6 months	CAPS-2
Goldstein et al. (2018) USA	47	38/9	47	Veterans, male and female, ages 18–69	46.8 ± 14.93	DSM-IV, full or subthreshold PTSD. (Structured Clin- ical Interview for DSM- IV-TR Axis I Disorders)	WL waiting list control group	lE Integrated Exercise (Aerobic, Resistance Yoga) 1 h 3 times per week	Independent	12 weeks	CAPS
Hall et al. (2020) USA	54	49/5	48	Military veterans, men and women aged *≥* 60 years,	T67.7 ± 3.2 C66.9 ± 4.3	DMS-V, meet- ing diagnostic criteria for PTSD.	Routine care	Aerobics, strength, balance and flexibility 60–90 min per session 3 times per week	Independent	12 weeks	PCL-5
Huberty et al. (2020) USA	90	0/90	48	Recruited through nonprofit stillbirth-related organizations	NR	DSM-IV, meeting diagnostic criteria for PTSD. (IES-R)	Attention Placebo	Yoga,personal. Hatha-basedyoga videos from an online streaming platform low dose: 60 min/week Medium dose: 150 min/week	Independent	12 weeks	IES-R
Jindani et al. (2015) Canada	80	9/71	50	community, men and women.	41.0 (range18-64)	DSM-IV. (PCL-17 > 57)	Waitlist	Yoga, group. Kundalini Yoga 90 min	Adjunctive Psychotherapy, excluding meditation	8weeeks	PCL-17
Mitchell et al. (2014) USA	38	0/38	38	Women (9 veterans, 29 civilians), ages 18–65, recruited from the U. S. VA Medical Center and Craigslist.	44.4 ± 12.4	DSM-IV, full or subthreshold PTSD diagnosis during in-person interview.	Assessment only control	Yoga, Group. Trauma Sensitive Yoga and Kripalu Yoga (Hatha Yoga). 12 weeks/week Once a week, or 6 weeks, x 2 weeks	Adjunctive Psychotherapy or medication	12 weeks	PCL-C
Nordbrandt et al. (2020) Denmark	318	150/168	213	18 years or above; recognised as a refugee or family reunified with a refugee;	44.6 ± 10.3	ICD-10, clinically diagnosed by medical doctor.	Treatment as usual (TAU)	Conventional Therapy (TAU) + Mixed physical activity 1 h Once a week	Adjunctive Psychotherapy or medication	20 weeks	HTQ
Reinhardt et al. (2018) USA	51	47/4	15	Military, male and female, ≥18 years old, active duty and veterans,	45.4 ± 13.3	DSM-IV, PTSD diagnosis. (SCID-CT)	Placed on waitlist	Yoga, group. Kripalu Yoga 2 × 90 min/week + family sessions	Adjunctive Psychotherapy or medication	10 weeks	CAPS
Rosenbaum et al. (2015a) Australia	81	68/13	58	81in-patients from thePTSD in-patient	47.8 ± 12.1	DSM-IV, psychiatrist- confirmed primary PTSD. (PCL-C > 45)	Conventional treatment	Resistance training 30 min 3 times a week	Adjunctive Psychotherapy + Medication + Group Therapy	12 weeks	PCL-C
Wolf et al. (2024) German	400	106/284 9unknown sex	323	Outpatients with mental illness	42.2 ± 13.3	symptoms of PTSD assessed with the PTSD Checklist for DSM-5	Conventional treatment	ImPuls (outdoor exercise, resistance training Transdiagnostic Group Exercise Intervention) + conventional treatment At least 30 min Moderate intensity Twice a week Total 90 min of exercise per week	Adjunctive Psychotherapy or medication	6 months	PCL-5
van der Kolk et al. (2014) USA	64	0/64	64	community, female, aged 18–58 years, recruited from commercials, research sites, and mental health professionals	42.9 ± 12.1	DSM-IV, meeting diagnostic criteria for PTSD. (CAPS > 45)	Attention placebo (health education program)	Yoga, Group. Trauma sessions informed by Hatha Yoga sessions 60 min	Adjunctive Psychotherapy or medication	10 weeks	CAPS
Whitworth et al. (2019a) USA	30	8/22	24	Community, men and women, age 18–45 years, non-treatment- seeking adults who screened positive for PTSD	29.10 ± 7.38	DSM-V, positive screen for PTSD and anxiety. (PDS5)	Non-exercise related educational activities	High intensity resistance exercise 30 min 3 times a week	Independent	3 weeks	PDS5
Whitworth et al. (2019b) USA	22	4/18	22	Participants were recruited from the local community using online classified listings (e.g., Craigslist), flyers,	33 ± 13.3	Posttraumatic Stress Diagnostic Scale for DSM-5	Attention control Non-exercise activities	High-intensity resistance training 5-min warm-up on stationary bike, 5 resistance training exercises 30 min 3 times a week	Independent	3 weeks	PDS5
Young-McCaughan et al. (2022) USA	72	66/6	36	Active duty service members seeking treatment for PTSD symptoms at Carl R. Darnall	36 ± 7.4	total scores ranging from 17 to 85, where higher scores indicate greater PTSD symptoms	Self-care interventions	Exercise 20–25 min 5 times a week	Independent	8 weeks	PCL-5

DSM = Diagnostic and Statistical Manual of Mental Disorders; CAPS=Clinician Administered PTSD Scale; CAPS-2 = Clinician-Administered PTSD Scale, Second Edition; HTQ = Harvard Trauma Questionnaire; IES-R = Impact of Event Scale; PCL-C = Posttraumatic Stress Disorder Checklist–Civilian Version; PCL-17 = PTSD Checklist with 17 items; PCL-5 = PTSD Checklist for DSM; PDS5 = Posttraumatic Diagnostic Scale; PTSD = post-traumatic stress disorder; SCID-CT = Structured clinical interview for DSMIV-TR axis I disorders, clinical trials version; ICD-10 = International Classification of Diseases, 10th Revision.

#### 3.2.2 Characteristics of the intervention

The 14 included studies employed a range of exercise interventions, as detailed in Table 1. Specifically, yoga, as a gentle yet effective intervention, was featured in 5 of these articles; resistance training, known for its relevance and effectiveness, appeared in 3 articles. The remaining 6 articles covered a diverse range of interventions, including combined exercise, integrated exercise, and aerobic exercise. The frequency of exercise interventions in these studies varied, basically following a 3-times-per-week schedule, and the intensity of each intervention was generally maintained at 30 to 60 min. In terms of the intervention period, most of the studies chose a time span of 3 weeks to 12 weeks, and it is worth mentioning that two of the studies even carried out continuous interventions for up to 6 months to observe the long-term effects of exercise.

#### 3.2.3 Outcome indicators

Different PTSD assessment tools are employed for various populations across different studies. Clinical evaluations commonly utilize the CAPS and its updated version, CAPS-2. For patient self-assessment, the Posttraumatic Stress Disorder Checklist (PCL) series is primarily used, with PCL-C frequently applied to civilian populations. Meanwhile, the PDS-5 is mainly employed to evaluate the effectiveness of psychological stress coping strategies and does not directly measure PTSD symptoms. Additionally, both the IES-R and HTQ are self-report instruments: the IES-R focuses on assessing individuals’ subjective stress responses to traumatic events, while the HTQ is widely applied for evaluating post-traumatic reactions across cultural contexts. The IES-R comprises 22 items rated on a 0–4 point scale, covering three dimensions: avoidance, intrusive memories, and hyperarousal. Higher total scores indicate greater subjective distress related to the trauma. The Italian version of the IES-R demonstrated internal consistency reliability coefficients ranging from 0.72 to 0.83 across dimensions (Craparo et al., 2013). In this study, the Cronbach’s *α* coefficient for the total score reached 0.98. The HTQ is applicable for assessing diverse populations, including those with bipolar disorder and borderline personality disorder, and its factor structure comprises three dimensions similar to the IES-R. The results of this study indicate that the exercise intervention group showed greater improvement in PTSD symptoms compared to the control group.

### 3.3 Risk of bias

**Figure 2:**
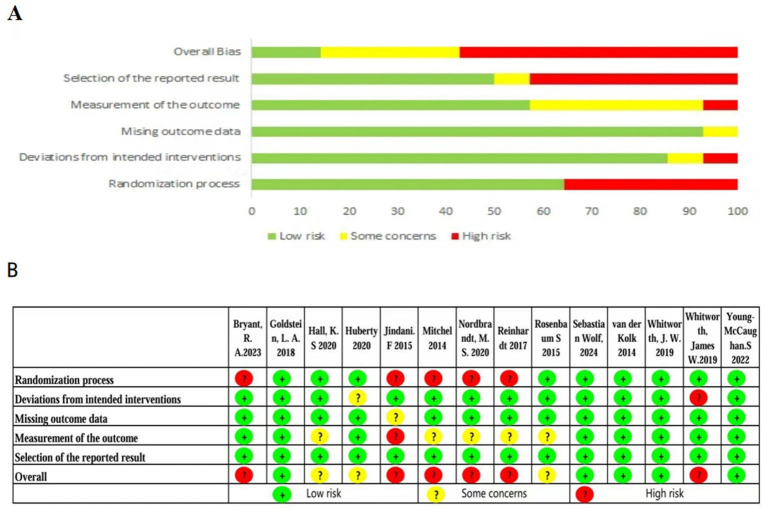
Quality Rating Scale for the Included Literature on the Efficacy of Exercise Interventions for PTSD. **(A)** Risk of bias in the included studies. **(B)** Risk of bias summary in the included studies.

The risk of bias assessment for the included studies is summarized in Figure 2. Judgments were made based on the pre-defined criteria outlined in the Methods section. Regarding the randomization process, 9 studies (64%) were rated as ‘low risk’ due to adequate sequence generation and allocation concealment. For deviations from the intended interventions, 5 studies (36%) were rated as ‘low risk’, primarily due to challenges in blinding participants and personnel in exercise interventions. Crucially, in the domain of ‘missing outcome data’ a point raised by the reviewer the majority of studies (12/14, 86%) were judged as ‘low risk’. This judgment was assigned because these studies either reported attrition rates below our pre-specified threshold of 20%, employed appropriate statistical methods to handle missing data (such as intention-to-treat analysis), or both. One study was rated ‘high risk’ due to a high dropout rate that was not adequately addressed, and one study provided insufficient information, leading to a judgment of ‘some concerns. In the measurement of the outcome, 8 studies (57%) that blinded the outcome assessors were rated ‘low risk’. All studies (100%) were rated ‘low risk’ for selective reporting, as all pre-specified outcomes were reported. The overall risk of bias assessment indicated that 5 studies (36%) had a low risk, 3 studies (21%) raised some concerns, and 6 studies (43%) had a high risk of bias.

## 4 Meta-analysis

**Figure 3:**
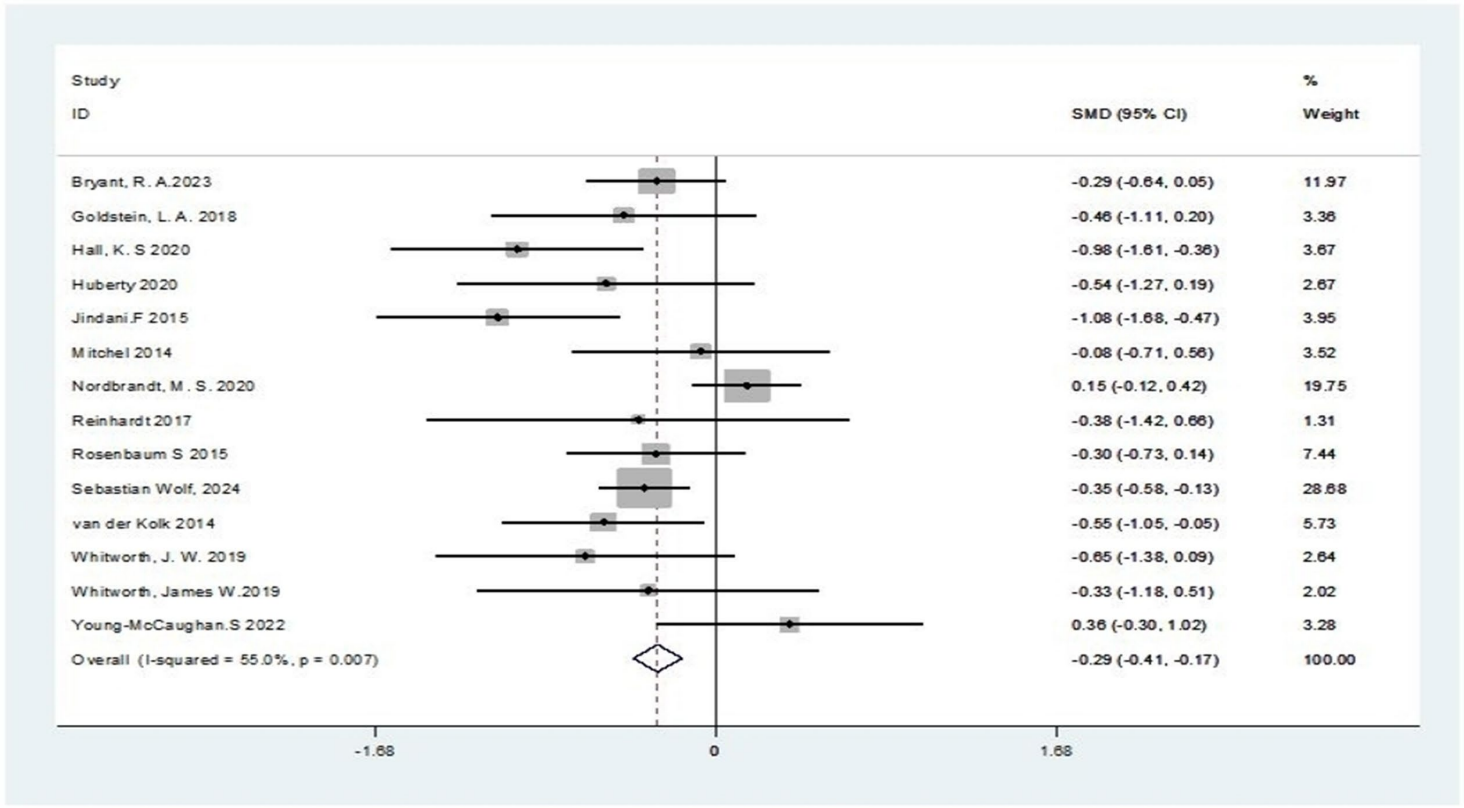
A test of heterogeneity in the efficacy of exercise interventions for PTSD.

**Figure 4:**
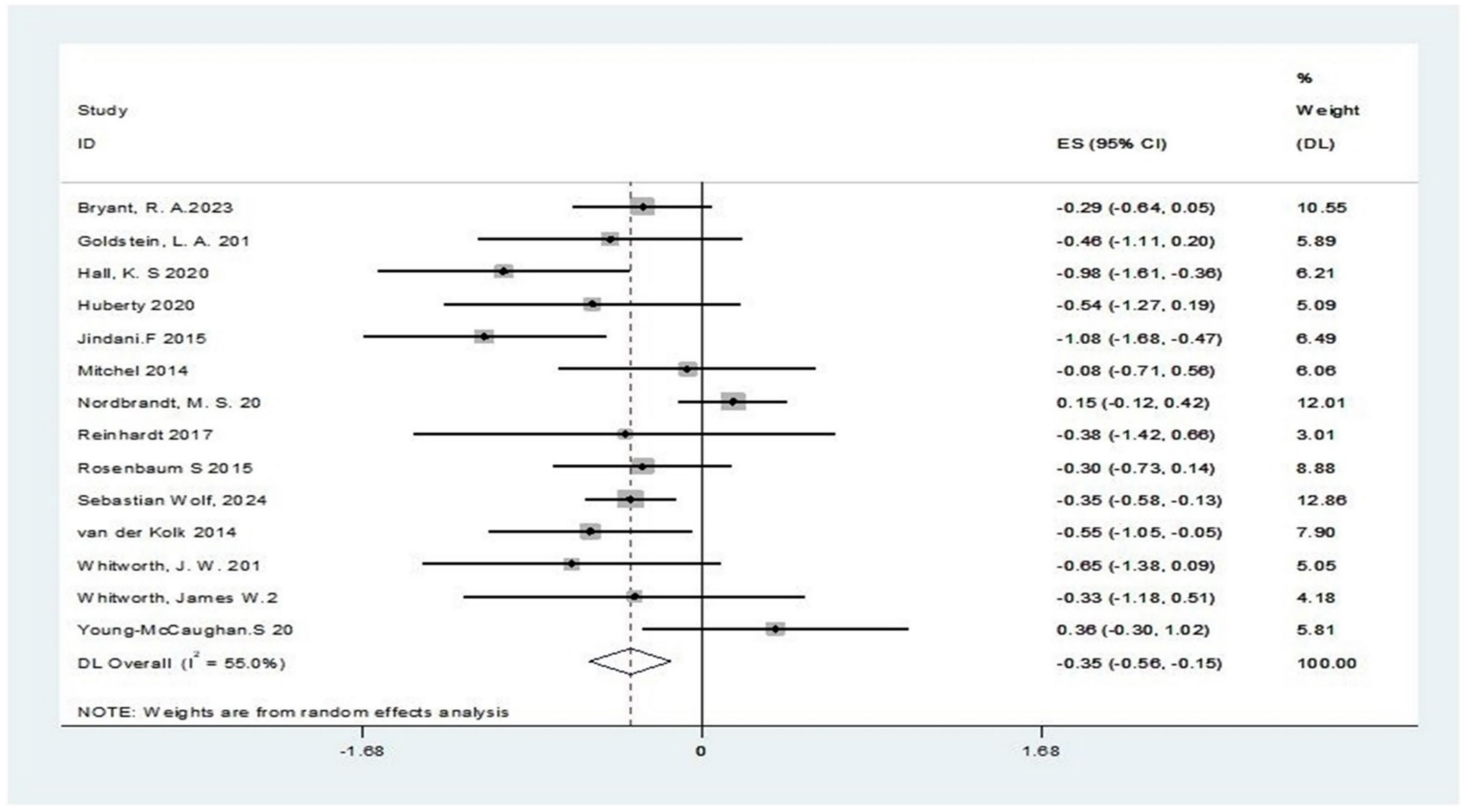
Combined random-effects results of the efficacy of exercise interventions for PTSD.

This research involved performing a diversity analysis on 14 pertinent studies, as depicted in Figure 3. The findings revealed a 55% I^2^ value surpassing the 50% mark, and a *p*-value below 0.1 in the Q-test, signifying notable diversity across the literature. This heterogeneity may be due to the non-uniformity of the output outcome metrics among the studies. Consequently, we opted to perform a meta-analysis employing a random-effects model, with its outcomes displayed in Figure 4. The pooled analysis demonstrated that, compared to the control group, the exercise intervention group showed a statistically significant reduction in PTSD symptoms, with a standardized mean difference (SMD) of −0.35 (95% CI: −0.56 to −0.15). The variance observed was of statistical importance (*p* < 0.05), as detailed in Table 2.

**Table 2:** Random effects significance test.

_ES	Exp (b)	Std. Err	t	P > t	[95%conf. [Interval]
_CONS	_700279	_0773434	−3.40	0.005	0.5563211, 0.8783309

## 5 Sensitivity analysis

**Figure 5:**
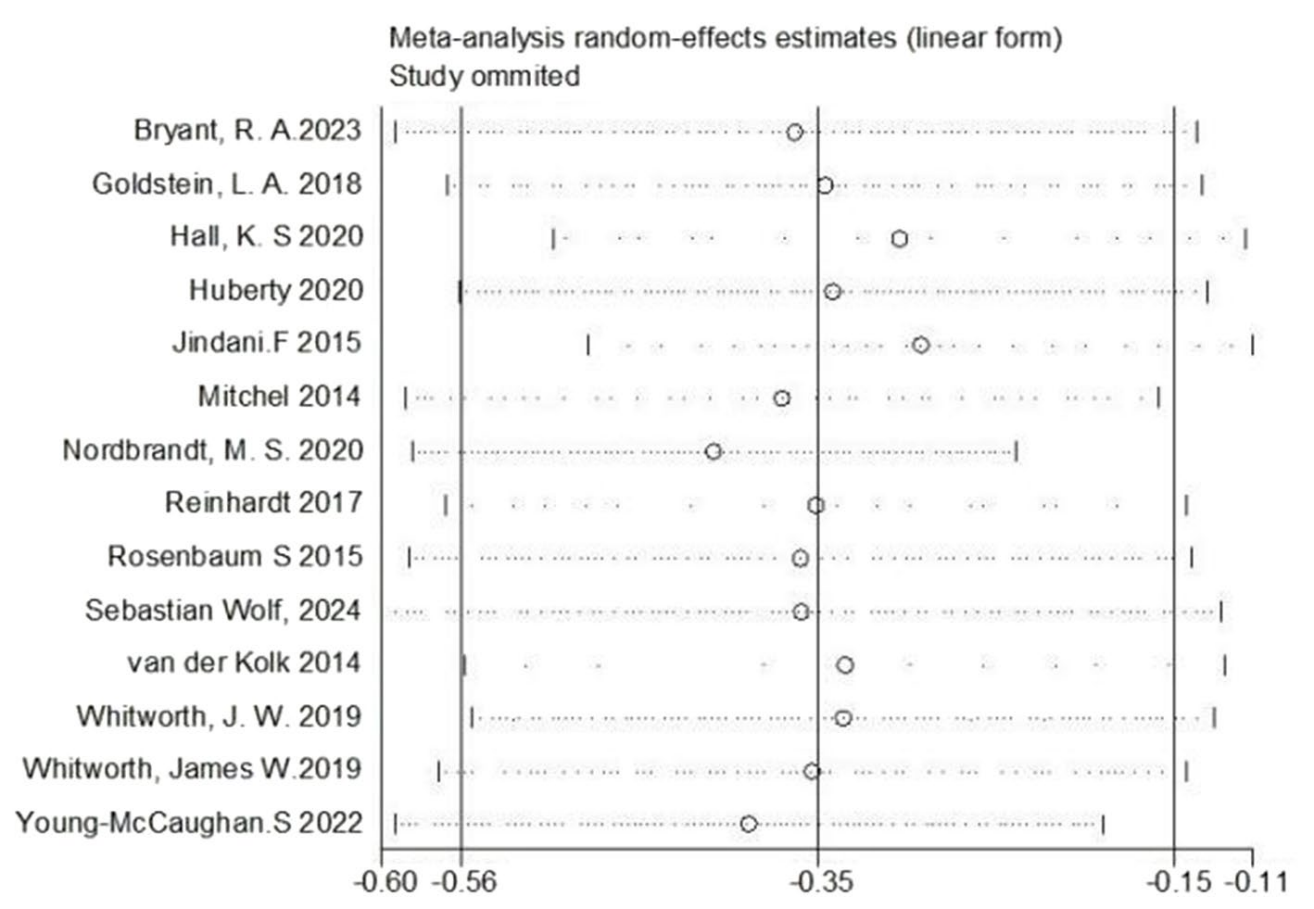
Sensitivity analysis of random effects of exercise intervention on the efficacy of PTSD.

Our research involved conducting a sensitivity analysis on the 14 articles incorporated. This test was performed by excluding each article individually and found that the combined effect sizes remained largely stable without significant fluctuations. This implies the robustness of the meta-analysis outcomes, indicating that no individual study significantly influenced the aggregate results, as depicted in Figure 5.

## 6 Bias situation

**Figure 6:**
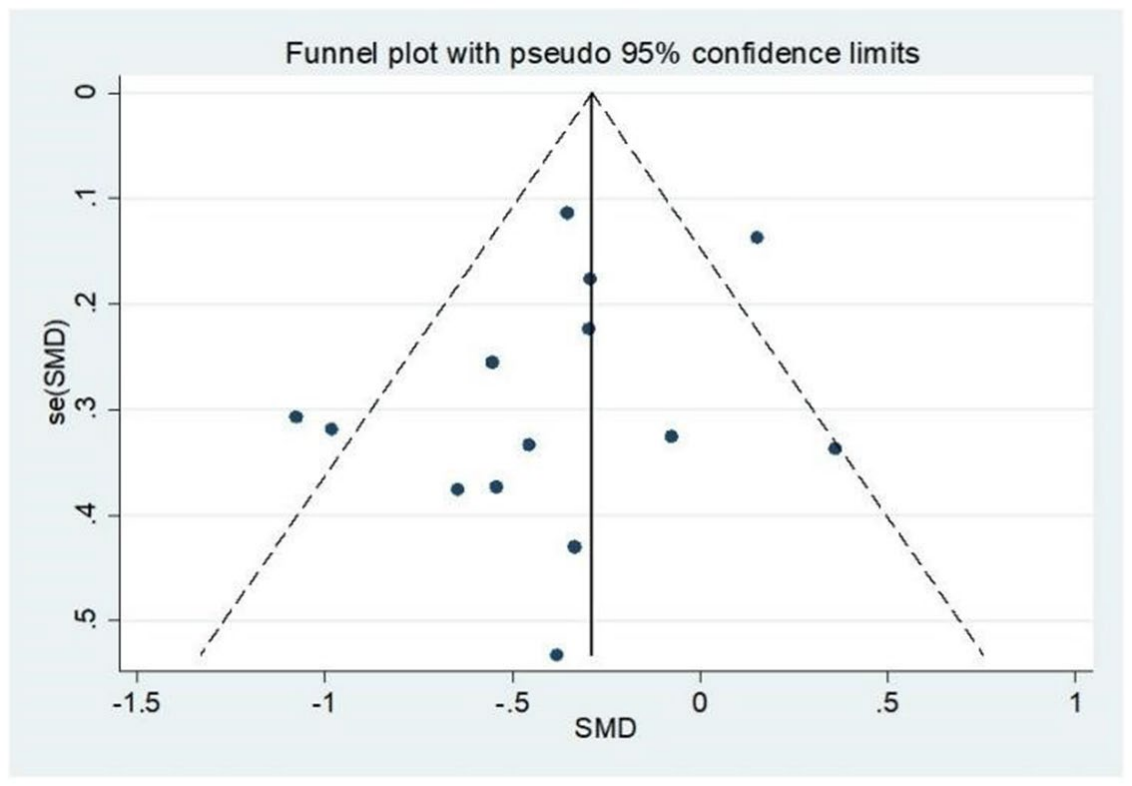
Funnel plot of the efficacy of exercise interventions for PTSD.

This study demonstrates that the funnel plot (Figure 6) indicates a high degree of symmetry in the data. Furthermore, a bias test was performed on the literature, yielding a result of *p* = 0.225, which exceeds the significance threshold of 0.05. The research indicates an absence of publication bias within the encompassed literature. This result improves the reliability and credibility of the study’s findings (Table 3).

**Table 3:** A test for bias in the efficacy of exercise interventions for PTSD Egger’s test.

Std Eff	Coef.	Std Err.	t	P > t	[95% Conf. Interval]
Slope	−0.055	0.202	−0.27	0.790	−0.49, 0.39
Bias	−1.134	0.897	−1.28	0.225	−3.06, 0.80

## 7 Subgroup analysis

An exhaustive subgroup analysis of multiple dimensions of exercise interventions was systematically conducted by the current study, including intervention programs, duration, frequency, intensity, and intervention groups. Varying degrees of heterogeneity among the subgroups were identified in the process. This series of subgroup analyses aims to enhance understanding of exercise interventions in treating PTSD patients and to investigate potential differences in intervention effects across various characteristics. This will enable the precise development of exercise intervention strategies for patients with PTSD. The subsequent section provides a description of the subgroup analysis pertaining to PTSD (Table 4).

**Table 4:** Integration of subgroup analyses of the efficacy of exercise interventions in PTSD.

Subgroup	Included studies	Heterogeneity	Test	Result (SMD)	*p*
		I^2^	*p*	95%*CI*	
Projects					
Other	6	72.4%	*p* = 0.003	−0.21 (−0.35, −0.06)	*p* = 0.004
Yoga	5	22.5%	*p* = 0.271	−0.56 (−0.85, −0.27)	*p* < 0.001
Resistance training	3	0.0%	*p* = 0.722	−0.38 (−0.72, −0.03)	*p* = 0.031
Time					
20 weeks≥	4	76.8%	*p* = 0.013	−0.18 (−0.33, −0.02)	*p* = 0.025
12 weeks	5	13.6%	*p* = 0.328	−0.44 (−0.70, −0.17)	*p* = 0.001
12 weeks<	6	52.1%	*p* = 0.054	−0.48 (−0.76, −0.21)	*p* = 0.001
Frequency					
3 times>	2	66.3%	*p* = 0.085	−0.15 (−0.40, 0.15)	*p* = 0.329
3 times	5	0.0%	*p* = 0.450	−0.51 (−0.78, −0.23)	*p* < 0.001
3 times<	7	68.5%	*p* = 0.004	−0.26 (−0.40, −0.11)	*p* = 0.001
Intensity					
30 min<	2	66.3%	*p* = 0.085	−0.15 (−0.46, 0.16)	*p* = 0.329
60 min>	8	70.6%	*p* = 0.001	−0.27 (−0.45, −0.09)	*p* = 0.004
30 min	6	0.0%	*p* = 0.881	−0.36 (−0.55, −0.17),	*p* < 0.001
Group					
Other	7	66.6%	*p* = 0.006	−0.25 (−0.39, −0.12)	*p* < 0.001
Veteran	4	64.5%	*p* = 0.038	−0.38 (−0.73, −0.03),	*p* = 0.033
Female	3	0.0%,	*p* = 0.472	−0.41 (−0.76, −0.06),	*p* = 0.020

**Figure 7:**
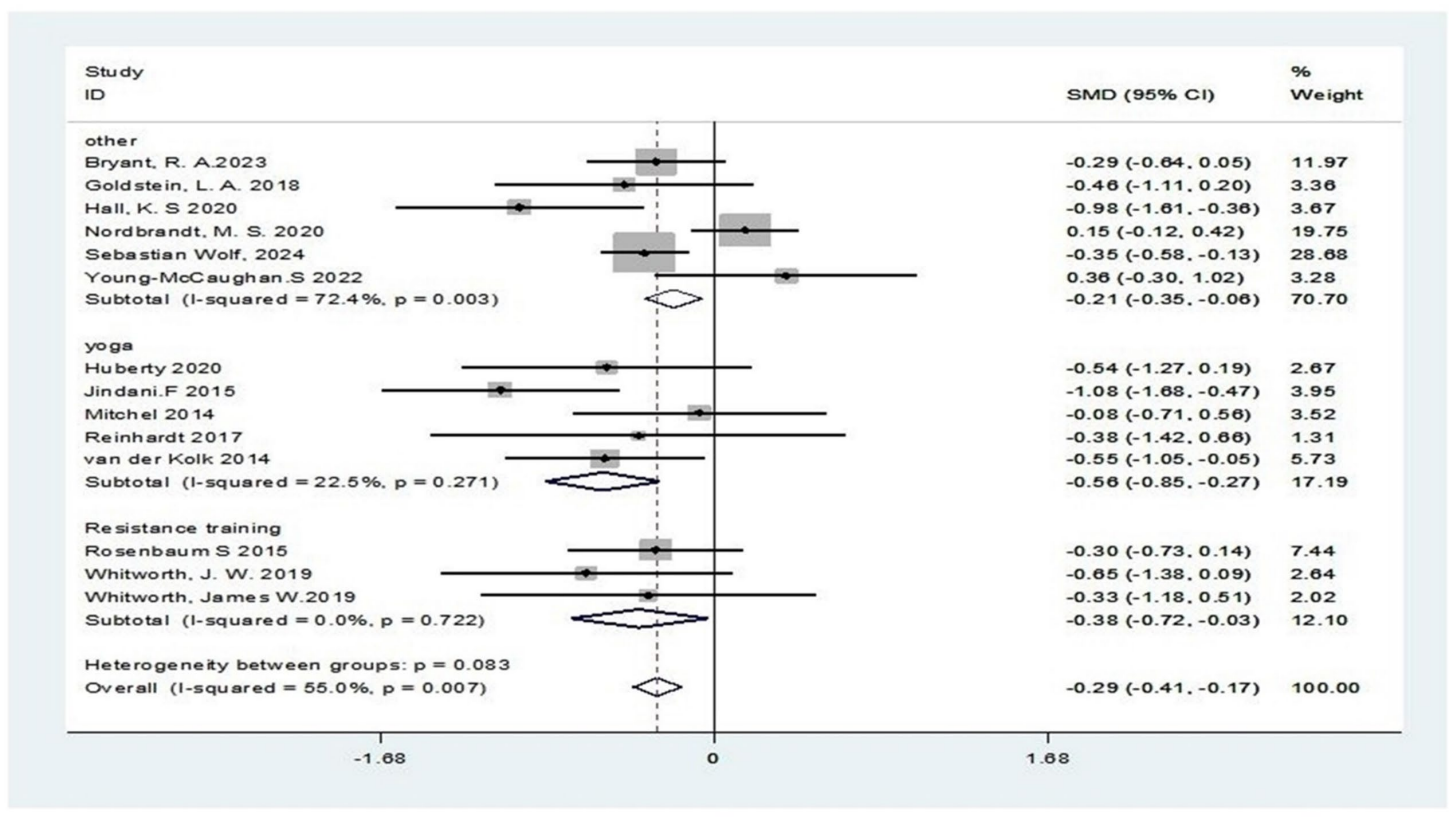
Subgroup analysis based on program of exercise intervention.

Subgroup analysis based on intervention type revealed that the heterogeneity index for yoga interventions (I^2^ = 22.5%) indicated low heterogeneity among studies. The standardized mean difference (SMD) was −0.56 (95%CI: −0.85 to −0.27), a statistically significant result (*p* < 0.001) exceeding the prespecified threshold for clinical significance (SMD ≥ 0.5). The heterogeneity index (I^2^) for resistance training interventions was 0%, indicating no heterogeneity. The standardized mean difference was −0.38 (95%CI: −0.72 to −0.03), with a Z-value of 2.16 (*p* = 0.033). This indicates resistance training also has therapeutic effects on PTSD symptoms, though its significance is lower than yoga’s, and the effect size is small, failing to reach the clinical significance threshold (SMD < 0.5). Its clinical significance may be limited. For other exercise programs (aerobic exercise, combined exercise, mixed exercise), the heterogeneity index (I^2^) reached 72.4%, indicating significant heterogeneity. The standardized mean difference was −0.21 (95% CI: −0.35 to −0.06), with a Z-score of 2.84 (*p* = 0.004). This indicates that while these therapies show statistical significance in reducing PTSD symptoms, their impact is relatively limited. With an effect size below 0.2, further research is needed to assess their clinical relevance (Figure 7).

**Figure 8:**
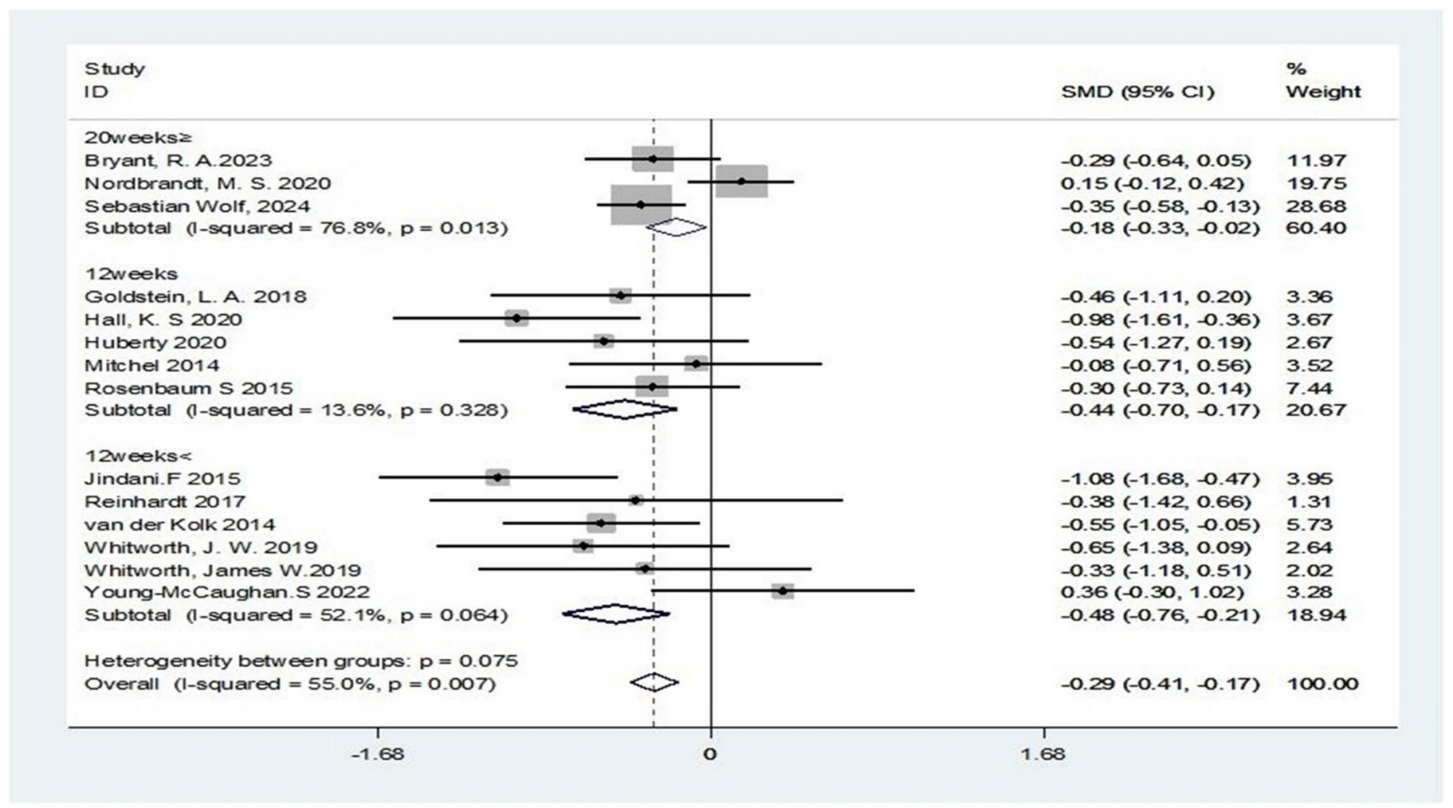
Subgroup analysis according to duration of intervention.

Second, in subgroup analyses stratified by intervention duration, studies implementing the 12-week program demonstrated lower heterogeneity (I^2^ = 13.6%). A significant moderate effect size was observed (SMD = −0.44; 95% CI: −0.70 to −0.17; Z = 3.26, *p* = 0.001), indicating statistically significant superior efficacy of the 12-week intervention over other durations in improving PTSD symptoms. This effect size approached 0.5, representing a small to moderate effect. Interventions shorter than 12 weeks showed moderate heterogeneity (I^2^ = 52.1%) but still yielded a significant effect size (SMD = −0.48; 95% CI: −0.76 to −0.21; Z = 3.43, *p* = 0.001). Despite moderate heterogeneity, the statistically significant outcome (*p* < 0.05) and relatively large effect size approaching 0.5, indicating a small to moderate effect demonstrate that short-term interventions provide clinically meaningful benefits for PTSD patients. The subgroup receiving extended intervention (≥20 weeks) showed significant heterogeneity (I^2^ = 77%), yet statistical significance persisted (SMD = −0.18; 95% CI: −0.33 to −0.02; Z = 2.34, *p* = 0.025), and the effect size remained below the threshold for clinical significance (SMD < 0.5). This diminishing effect size suggests that extending intervention duration in PTSD management leads to diminishing returns (Figure 8).

**Figure 9:**
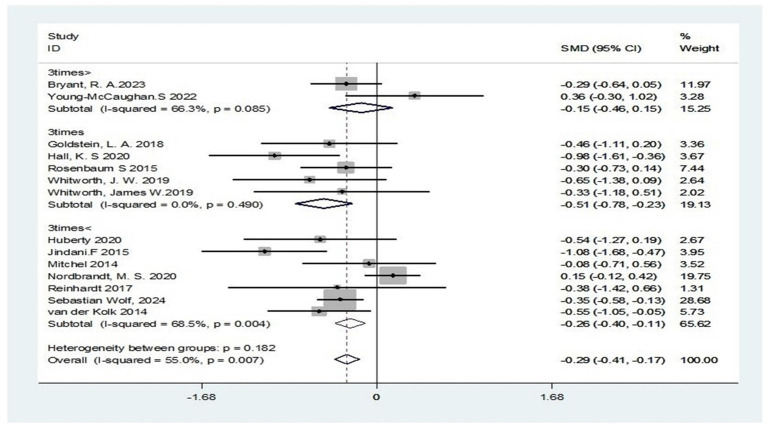
Subgroup analysis based on frequency of exercise intervention.

Additionally, subgroup analyses stratified by intervention frequency revealed complete homogeneity across studies for the three-times-weekly regimen (I^2^ = 0%). Moderate to large effect sizes were observed (SMD = −0.51; 95% CI: −0.78 to −0.23; Z = 3.64, *p* < 0.001), indicating that the three-times-weekly intervention was not only statistically significant (*p* < 0.001) but also substantially exceeded our prespecified threshold for clinical significance (SMD ≥ 0.5). The subgroup with fewer than three weekly sessions exhibited significant heterogeneity (I^2^ = 69%). Although the effect remained statistically significant (SMD = −0.26; 95% CI: −0.40 to −0.11; Z = 3.39, *p* = 0.001), the reduced effect size compared to the three-times-weekly regimen suggests diminished efficacy at lower frequencies. Interventions exceeding three sessions per week exhibited significant heterogeneity (I^2^ = 66.3%). The non-significant result (SMD = −0.15; 95% CI: −0.40 to 0.15; Z = 0.98, *p* = 0.329), combined with substantial heterogeneity and a small effect size, suggests a marginal diminishing returns trend for high-frequency interventions in PTSD treatment (Figure 9).

**Figure 10:**
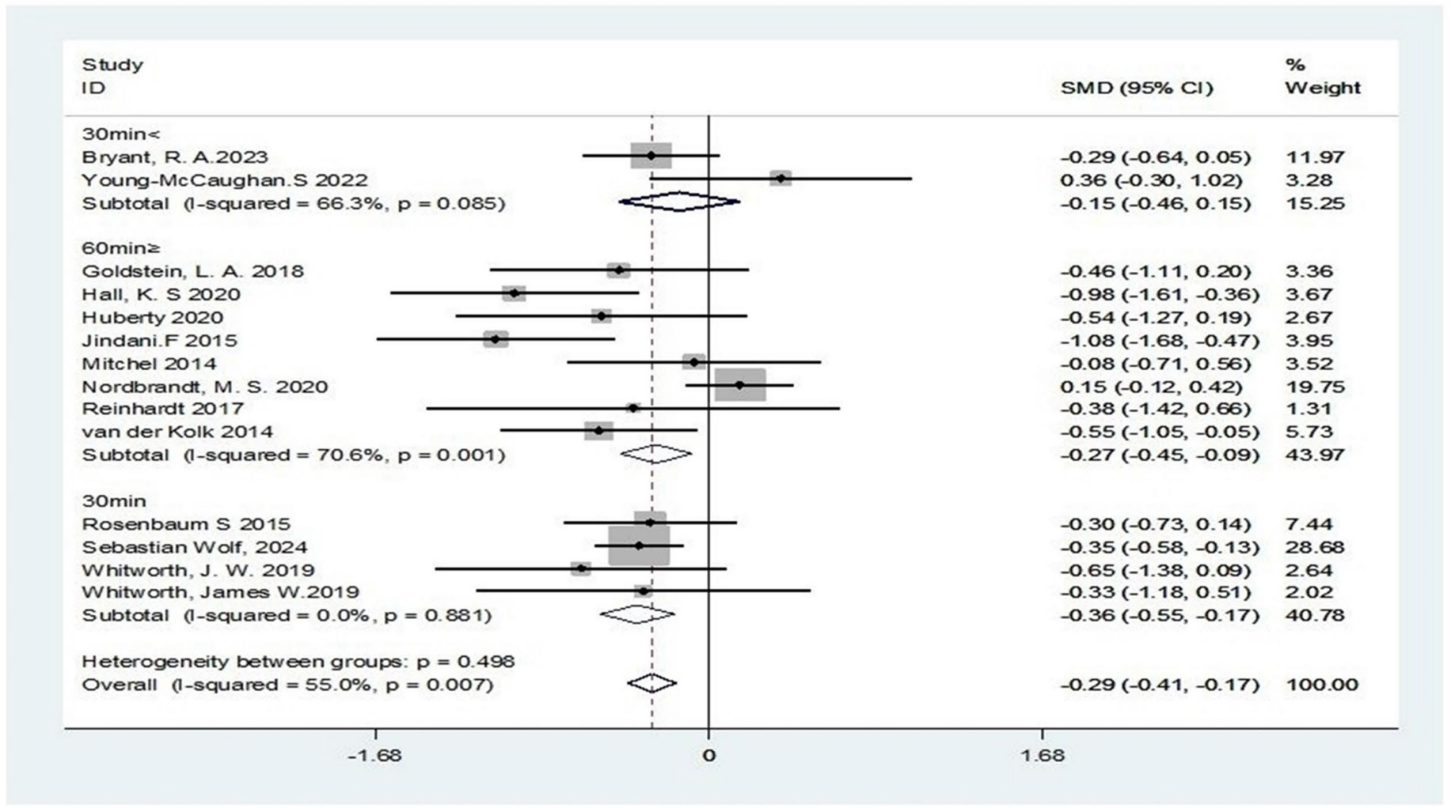
Subgroup analysis based on exercise intervention intensity.

Additionally, subgroup analysis focusing on interventions with a precise 30-min duration revealed complete homogeneity across studies (I^2^ = 0%). A clinically meaningful effect was observed (SMD = −0.36; 95% CI: −0.55 to −0.17; Z = 3.78, *p* < 0.001), indicating that a 30-min session represents the optimal treatment duration for achieving statistically significant symptom improvement in PTSD management. However, this effect size falls within the small to moderate range, potentially limiting its clinical significance. (I^2^ = 71%). Despite methodological heterogeneity, a moderate yet significant therapeutic effect persisted (SMD = -0.27; 95% CI: −0.45 to −0.09; Z = 2.90, *p* = 0.004), suggesting extended treatment durations may retain partial efficacy in alleviating PTSD symptoms. Therapeutic durations below the threshold (30 min) exhibited significant heterogeneity (I^2^ = 66.3%). No significant therapeutic effect was observed (SMD = −0.15; 95% CI: −0.46 to 0.16; Z = 0.98, *p* = 0.329), suggesting that shortening treatment duration in PTSD protocols may result in suboptimal therapeutic outcomes (Figure 10).

**Figure 11:**
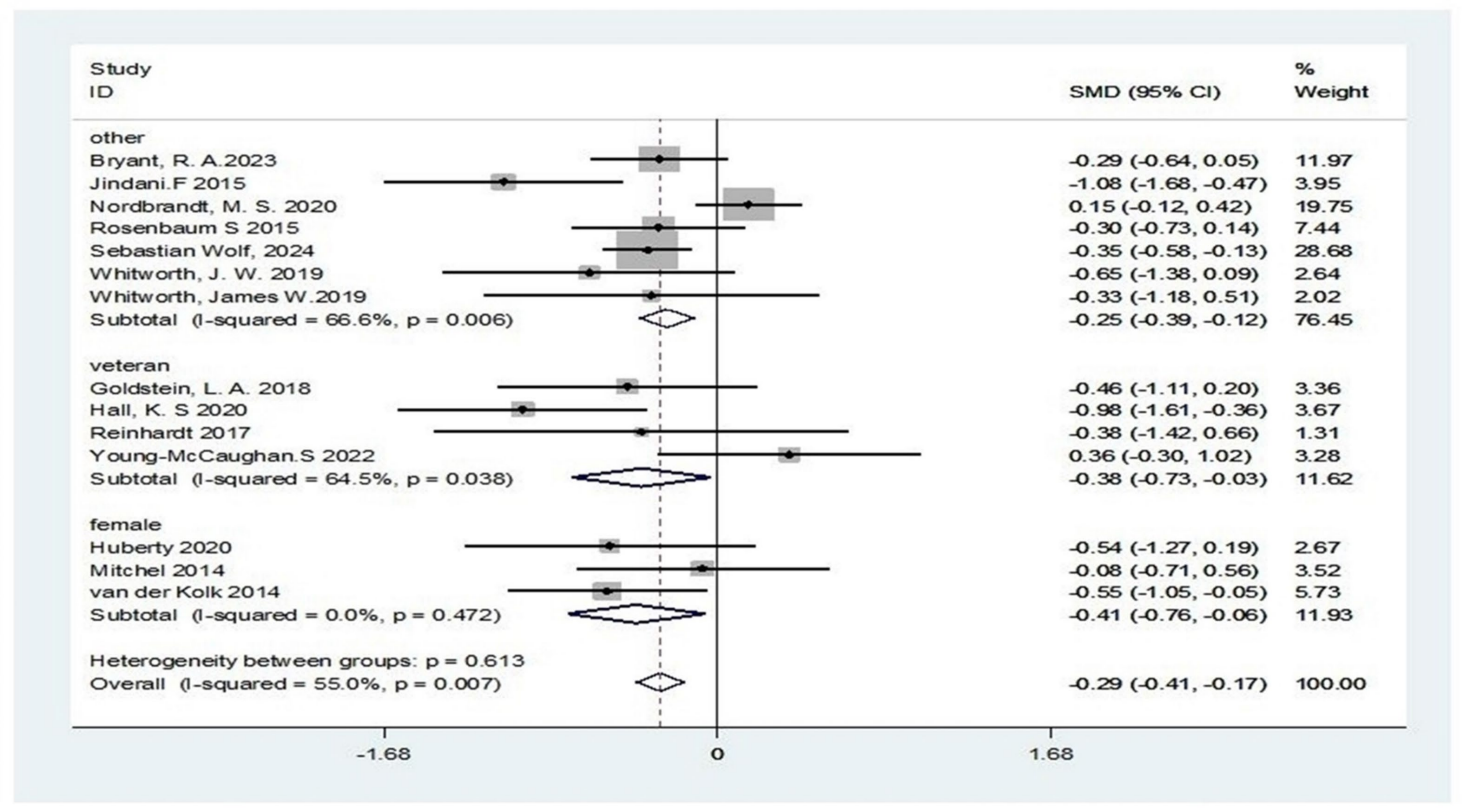
Subgroup analysis according to the group of exercise intervention.

Finally, subgroup analysis of the OTHER cohort revealed significant heterogeneity across studies (I^2^ = 67%). Nevertheless, a robust therapeutic effect was confirmed (standardized mean difference = −0.25; 95% CI: −0.39 to −0.12; Z = 3.65, *p* < 0.001), indicating clinically meaningful reduction in PTSD symptoms for this patient subgroup. The veteran subgroup analysis revealed moderate methodological variability (I^2^ = 65%). Despite this variability, a moderate effect size was observed (SMD = −0.38; 95% CI: −0.73 to −0.03; Z = 2.13, *p* = 0.033), suggesting individuals with combat exposure may derive significant benefit from this intervention. The female patient subgroup demonstrated excellent consistency of results (I^2^ = 0%) and clinically relevant effect size (SMD = −0.41; 95% CI: −0.76 to −0.06; Z = 2.32, *p* = 0.020), with an effect size approaching 0.5, indicating a small to moderate effect. This suggests the intervention demonstrates superior efficacy in alleviating PTSD symptoms among female populations (Figure 11).

## 8 Discussion

The meta-analysis, encompassing 14 randomized controlled trials (RCTs), demonstrated that exercise interventions provide a statistically significant and clinically meaningful reduction in PTSD symptoms compared to control conditions (SMD = −0.35; *p* < 0.05). Although the overall effect size is in the small-to-medium range, it is driven by specific interventions that showed large, clinically important effects. Significant variability among studies was noted (I^2^ = 55%), likely due to differences in trial methodologies, intervention protocols, population demographics, and evaluation tools Key heterogeneity sources included disparities in randomization procedures, blinding implementation, comparator group configurations, exercise modalities (e.g., yoga vs. aerobic training), dosage parameters (intensity/frequency/duration), population demographics, baseline symptom severity, and outcome measurement tools. To enhance result validity, subgroup analyses are recommended to examine differential effects across: (1) exercise modalities (yoga/aerobic/resistance), (2) dosage parameters (weekly frequency/session duration), (3) treatment duration (acute vs. maintenance phases), (4) population subgroups (gender/age/occupation), and (5) assessment instruments (CAPS/PCL-5/IES-R). Subgroup analyses demonstrated superior efficacy for mind–body (yoga) and strength-focused (resistance training) modalities, particularly when administered in 12-week protocols with thrice-weekly sessions (SMD = −0.44; *p* < 0.001). Protocols deviating from this frequency demonstrated attenuated efficacy, suggesting three sessions weekly represents an optimal therapeutic cadence.

Subsequent empirical research indicates that yoga significantly reduces the allostatic load in three principal stress response routes: the autonomic nervous system (ANS), hypothalamic–pituitary–adrenal (HPA) axis, and GABAergic system, providing substantial therapeutic benefits. Björkman and Ekblom’s (2022) research indicates that the therapeutic advantages of exercise as a supplementary intervention for PTSD may become more significant with increased exercise dosage. Additionally, Yuan’s et al. (2025) meta-analysis has reinforced the effectiveness of physical activity as a supplementary therapy for PTSD, showing its potential to ease fundamental PTSD symptoms, reduce concurrent anxiety and hopelessness, and enhance sleep quality. Although the study conducted by Yuan et al. (2025) identified yoga as the optimal exercise modality via subgroup analysis, recommending a frequency of 1–2 sessions per week (30–60 min per session) or 3–5 sessions weekly (30–45 min per session) the present investigation revealed through more comprehensive subgroup analyses that resistance training also demonstrates notable efficacy. Optimal outcomes for resistance training were observed when administered three times weekly, with sessions lasting 30–60 min over a 12-week intervention period. The therapeutic efficacy of yoga has been attributed to its integrative mind–body approach, which systematically combines controlled breathing, physical postures, muscle relaxation, and mindfulness meditation (Granath et al., 2006). Research indicates that this approach improves PTSD patients’ ability to endure upsetting feelings and handle stress (Brown and Gerbarg, 2005; Rocha et al., 2012; West et al., 2004; Streeter et al., 2012). These studies have repeatedly shown a robust connection between resistance training and notable enhancements in sleep quality, validating its efficacy in diminishing symptoms of anxiety and depression associated with post-traumatic stress disorder (PTSD) (Kovacevic et al., 2018). Furthermore, the significant anxiolytic effects observed in resistance training interventions are of critical clinical relevance for individuals with PTSD. Concurrent anxiety symptoms, along with impaired sleep quality, are recognized factors that can exacerbate the severity of PTSD (Whitworth et al., 2019a; Whitworth et al., 2019b).

This meta-analysis broadened the study population by including female participants, whereas previous research predominantly focused on male military and non-military cohorts. The findings suggest that physical activity programs may provide enhanced therapeutic benefits for women with PTSD. Epidemiological data reveal that in the United States, women are diagnosed with PTSD at approximately twice the rate of men (Kessler et al., 2005). Furthermore, epidemiological research suggests that women are not only more likely to develop PTSD after trauma but are also disproportionately exposed to specific types of traumatic events that carry a particularly high risk for the disorder, such as sexual assault and childhood abuse (Chapman et al., 2004; Corso et al., 2008; Edwards et al., 2003; Felitti et al., 1998; Kessler et al., 2013; Walker et al., 1999). A demographic of particular clinical concern is mothers who have experienced stillbirth, as this population frequently experiences profound psychological trauma due to the simultaneous occurrence of birth and fetal demise (Gold et al., 2016). Relative to the broader population, these people show a seven times higher chance of developing PTSD symptoms and a quadrupled probability of receiving a diagnosis of concurrent clinical depression in the postnatal phase. Furthermore, a twofold increase in the risk of being diagnosed with anxiety disorders was observed. Typical manifestations like frequent flashbacks, heightened alertness, increased alertness, overthinking, and evasive actions have been noted to continue for durations ranging from 2 months to 18 years in clinical groups (Huberty et al., 2020). In this study, a non-pharmacological intervention involving a structured yoga regimen comprising controlled breathing techniques, postural alignment exercises, and mindfulness meditation was utilized to ameliorate PTSD symptoms among the cohort of enrolled female participants (Iyengar, 2013). Yoga and resistance training have shown therapeutic benefits, in particular their significant effects on female patients. This concept of psychomotor therapy is based on the clinically valid body theory that structured physical exercise helps regulate emotions, restores bodily safety, and reduces hyperarousal: the core dysfunctions of PTSD. Future studies might incorporate assessments from psychomotor therapy to further understand how different exercise techniques help relieve symptoms in different populations.

Numerous constraints are associated with this research. Initially, the presence of heterogeneity bias, stemming from varying outcome metrics in randomized controlled trials (RCTs), is inevitable. Additionally, most of the studies examined featured brief intervention periods (3–12 weeks), leaving the prolonged effectiveness of exercise programs, especially in terms of their enduring effect on chronic PTSD symptoms, ambiguous. Furthermore, the absence of population-specific investigations (e.g., elderly individuals, children/adolescents, and culturally diverse groups) constrain the generalizability of the findings. Additional rigorously designed RCTs are required to comprehensively evaluate the mechanistic effects of exercise-based interventions on PTSD pathophysiology. Despite Egger’s test revealing no notable bias in publication, the possibility of finding unpublished negative results must not be overlooked.

In addition to the limitations previously noted, the potential influence of the exercise environment (e.g., outdoor natural settings versus indoor facilities) on intervention outcomes was not explored. A growing body of evidence indicates that “green exercise” physical activity conducted in natural environments may confer psychological benefits beyond those achieved indoors, such as more substantial reductions in stress and greater improvements in mood and well-being (Lawton et al., 2017; Menardo et al., 2021; Noseworthy et al., 2017). For individuals with PTSD, hypervigilance and avoidance symptoms may be more effectively alleviated through exercise conducted in safe and tranquil outdoor environments, while also offering restorative settings that are distanced from daily trauma cues. Among the included studies, only the transdiagnostic group exercise intervention implemented by Wolf et al. (2024) was explicitly conducted outdoors; the remaining interventions took place in indoor settings, such as gyms or clinics. The influence of the exercise environment on PTSD outcomes thus constitutes a promising and nuanced area for future research, which could inform the development of more precise and effective intervention protocols.

## 9 Implications for current practice

To summarize, the present data provides practical guidance for clinicians. The consistent benefits shown in multiple RCTs support the systematic integration of structured, supervised exercise programs into PTSD treatment. Together, mental health and exercise professionals can design customized protocols using yoga or resistance training depending on frequency and duration parameters chosen as most effective. This approach can make accessible low-risk therapies available to individuals with PTSD more accessible.

## 10 Future research directions

Future research should focus on three important directions. First, the effects of exercise programs on a larger range of physiological and psychological parameters should be studied. Second, direct comparisons of the effectiveness of different exercise methods and their application to different types of trauma should be considered. Third, variability in the effects of intervention should be investigated in order to develop personalized treatment plans. One direction is to study how core psychomotor therapy principles such as body awareness, movement sequencing, and nonverbal expression might be integrated into structured exercise programs for PTSD for better therapeutic specificity and clinical utility. Finally, as the context is understudied, the effect of the exercise environment should be studied. For example, comparisons of results from outdoor ‘green exercise’ and indoor settings could provide useful information on optimal interventions and patient engagement.

## 11 Conclusion

This systematic review and meta-analysis synthesizes current evidence for optimizing exercise-based interventions for PTSD. Exercise reduces severity significantly and clinically significant severity compared with control (SMD = −0.35; *p* < 0.05). Even though effect size is small to medium, some interventions have large effects.

To facilitate the translation of this evidence into practice, the following recommendations are proposed. First, structured exercise, particularly yoga or resistance training, should be considered as an evidence-based adjunctive intervention for adults with PTSD. An optimal protocol derived from this analysis involves sessions conducted three times per week for 30–60 min over a 12-week period. Second, intervention personalization is advised. For female patients, mind–body practices such as yoga are strongly recommended due to evidence suggesting potentially greater benefit. For veteran populations, resistance training or structured group programs, including those conducted in integrated or outdoor settings, may be prioritized to align with common preferences and enhance engagement. Third, regarding implementation context, while exercise is effective in standard indoor settings, the potential added value of ‘green exercise’ in outdoor natural environments could be explored to potentially augment stress reduction and address symptoms such as behavioral avoidance. Finally, exercise programs can be safely integrated with first-line psychotherapies (e.g., Prolonged Exposure, Cognitive Processing Therapy). This combination may improve overall treatment adherence and outcomes by incorporating a somatic, non-threatening component into trauma-focused care.

## Data Availability

The datasets presented in this study can be found in online repositories. The names of the repository/repositories and accession number(s) can be found in the article/supplementary material.

## References

[ref1] Björkman F. Ekblom Ö. (2022). Physical exercise as treatment for PTSD: a systematic review and meta-analysis. Mil. Med. 187, e1103–e1113. doi: 10.1093/milmed/usab497 34850063

[ref2] Brown R. P. Gerbarg P. L. (2005). Sudarshan Kriya yogic breathing in the treatment of stress, anxiety, and depression: part II-clinical applications and guidelines. J. Altern. Complement. Med. 11, 711–717. doi: 10.1089/acm.2005.11.711, 16131297

[ref3] Bryant R. A. Dawson K. S. Azevedo S. Yadav S. Cahill C. Kenny L. (2023). Augmenting trauma-focused psychotherapy for post-traumatic stress disorder with brief aerobic exercise in Australia: a randomised clinical trial. Lancet Psychiatry 10, 21–29. doi: 10.1016/S2215-0366(22)00368-6, 36436532

[ref4] Chapman D. P. Whitfield C. L. Felitti V. J. Dube S. R. Edwards V. J. Anda R. F. (2004). Adverse childhood experiences and the risk of depressive disorders in adulthood. J. Affect. Disord. 82, 217–225. doi: 10.1016/j.jad.2003.12.013, 15488250

[ref5] Charlson F. van Ommeren M. Flaxman A. Cornett J. Whiteford H. Saxena S. (2019). New WHO prevalence estimates of mental disorders in conflict settings: a systematic review and meta-analysis. Lancet 394, 240–248. doi: 10.1016/S0140-6736(19)31141-9, 31200992 PMC6657025

[ref6] Corso P. S. Edwards V. J. Fang X. Mercy J. A. (2008). Health-related quality of life among adults who experienced maltreatment during childhood. Am. J. Public Health 98, 1094–1100. doi: 10.2105/AJPH.2007.119826, 18445797 PMC2377283

[ref9001] Craparo G. Faraci P. Rotondo G. Gori A. (2013). The impact of event scale - revised: psychometric properties of the Italian version in a sample of flood victims. Neuropsychiatr Dis Treat. 9, 1427–1432. doi: 10.2147/NDT.S51793 24092980 PMC3788700

[ref7] Edwards V. J. Holden G. W. Felitti V. J. Anda R. F. (2003). Relationship between multiple forms of childhood maltreatment and adult mental health in community respondents: results from the adverse childhood experiences study. Am. J. Psychiatry 160, 1453–1460. doi: 10.1176/appi.ajp.160.8.1453, 12900308

[ref8] Felitti V. J. Anda R. F. Nordenberg D. Williamson D. F. Spitz A. M. Edwards V. (1998). Relationship of childhood abuse and household dysfunction to many of the leading causes of death in adults: the adverse childhood experiences (ACE) study. Am. J. Prev. Med. 14, 245–258. doi: 10.1016/S0749-3797(98)00017-8, 9635069

[ref9] Foa E. B. Hembree E. A. Rothbaum B. O. (2007). Prolonged exposure therapy for PTSD: Emotional processing of traumatic experiences. Therapist guide. New York: Oxford University Press.

[ref12] Garber C. E. Blissmer B. Deschenes M. R. Franklin B. A. Lamonte M. J. Lee I. M. (2011). American College of Sports Medicine position stand. Quantity and quality of exercise for developing and maintaining cardiorespiratory, musculoskeletal, and neuromotor fitness in apparently healthy adults: guidance for prescribing exercise. Med. Sci. Sports Exerc. 43, 1334–1359. doi: 10.1249/MSS.0b013e318213fefb, 21694556

[ref13] Gold K. J. Leon I. Boggs M. E. Sen A. (2016). Depression and posttraumatic stress symptoms after perinatal loss in a population-based sample. J. Women's Health 25, 263–269. doi: 10.1089/jwh.2015.5284, 26258870 PMC4955602

[ref14] Goldstein L. A. Mehling W. E. Metzler T. J. Cohen B. E. Barnes D. E. Choucroun G. J. (2018). Veterans group exercise: a randomized pilot trial of an integrative exercise program for veterans with posttraumatic stress. J. Affect. Disord. 227, 345–352. doi: 10.1016/j.jad.2017.11.002, 29145076

[ref15] Granath J. Ingvarsson S. von Thiele U. Lundberg U. (2006). Stress management: a randomized study of cognitive behavioural therapy and yoga. Cogn. Behav. Ther. 35, 3–10. doi: 10.1080/16506070500401292, 16500773

[ref16] Hall K. S. Gregg J. J. Bosworth H. B. Beckham J. C. Hoerster K. D. Sloane R. (2020). Pilot randomized controlled trial of exercise training for older veterans with PTSD. J. Behav. Med. 43, 648–659. doi: 10.1007/s10865-019-00073-w, 31264055 PMC6938572

[ref17] Hedges L. V. Olkin I. (1985). Statistical methods for Meta-analysis. San Diego, CA: Academic Press.

[ref18] Hegberg N. J. Hayes J. P. Hayes S. M. (2019). Exercise intervention in PTSD: a narrative review and rationale for implementation. Front. Psych. 10:133. doi: 10.3389/fpsyt.2019.00133, 30949075 PMC6437073

[ref19] Huberty J. Sullivan M. Green J. Kurka J. Leiferman J. Gold K. (2020). Online yoga to reduce post traumatic stress in women who have experienced stillbirth: a randomized control feasibility trial. BMC Complement. Med. Ther. 20:353. doi: 10.1186/s12906-020-02926-3, 32503517 PMC7275350

[ref20] Iyengar B. K. S. (2013). BKS Iyengar yoga: The path to holistic health. London: Dorling Kindersley.

[ref21] Jindani F. Turner N. Khalsa S. B. S. (2015). A yoga intervention for posttraumatic stress: a preliminary randomized control trial. Evid. Based Complement. Alternat. Med. 2015:351746. doi: 10.1155/2015/351746, 26366179 PMC4558444

[ref22] Kessler R. C. Berglund P. Demler O. Jin R. Merikangas K. R. Walters E. E. (2005). Lifetime prevalence and age-of-onset distributions of DSM-IV disorders in the National Comorbidity Survey Replication. Arch. Gen. Psychiatry 62, 593–602. doi: 10.1001/archpsyc.62.6.593, 15939837

[ref23] Kessler R. C. Sonnega A. Bromet E. Hughes M. Nelson C. B. (2013). “Posttraumatic stress disorder in the National Comorbidity Survey” in Fear and anxiety. ed. Rosen G. M. (New York: Routledge), 22–34. 10.1001/archpsyc.1995.03950240066012 7492257

[ref24] Kim S. H. Schneider S. M. Kravitz L. Mermier C. Burge M. R. (2013). Mind-body practices for posttraumatic stress disorder. J. Investig. Med. 61, 827–834. doi: 10.2310/JIM.0b013e3182906862, 23609463 PMC3668544

[ref25] Koch S. C. Riege R. F. F. Tisborn K. Biondo J. Martin L. Beelmann A. (2019). Effects of dance movement therapy and dance on health-related psychological outcomes. A meta-analysis update. Front. Psychol. 10:1806. doi: 10.3389/fpsyg.2019.01806, 31481910 PMC6710484

[ref26] Kovacevic A. Mavros Y. Heisz J. J. Fiatarone Singh M. A. (2018). The effect of resistance exercise on sleep: a systematic review of randomized controlled trials. Sleep Med. Rev. 39, 52–68. doi: 10.1016/j.smrv.2017.07.002, 28919335

[ref27] Kysar-Moon A. Vasquez M. Luppen T. (2021). Trauma-sensitive yoga interventions and posttraumatic stress and depression outcomes among women: a systematic review and analysis of randomized control trials. Int. J. Yoga Ther. 31:23. doi: 10.17761/2021-D-20-00005 33543258

[ref28] Laplaud N. Perrochon A. Gallou-Guyot M. Moens M. Goudman L. David R. (2023). Management of post-traumatic stress disorder symptoms by yoga: an overview. BMC Complement. Med. Ther. 23:258. doi: 10.1186/s12906-023-04074-w, 37480017 PMC10360332

[ref29] Lawton E. Brymer E. Clough P. Denovan A. (2017). The relationship between the physical activity environment, nature relatedness, anxiety, and the psychological well-being benefits of regular exercisers. Front. Psychol. 8:1058. doi: 10.3389/fpsyg.2017.01058, 28694788 PMC5483473

[ref30] Leppink J. O’Sullivan P. Winston K. (2016). Effect size-large, medium, and small. Perspect Med Educ. 5, 347–349. doi: 10.1007/s40037-016-0308-y, 27752936 PMC5122517

[ref31] Martin A. Naunton M. Kosari S. Peterson G. Thomas J. Christenson J. K. (2021). Treatment guidelines for PTSD: a systematic review. J. Clin. Med. 10:4175. doi: 10.3390/jcm10184175, 34575284 PMC8471692

[ref9002] Martinez-Calderon J. Villar-Alises O. García-Muñoz C. Pineda-Escobar S. Matias-Soto J. (2024). Multimodal exercise programs may improve posttraumatic stress disorders symptoms and quality of life in adults with PTSD: an overview of systematic reviews with meta-analysis. Clin. rehabil. 38, 573–588. doi: 10.1177/02692155231225466 38258461

[ref32] Menardo E. Brondino M. Hall R. Pasini M. (2021). Restorativeness in natural and urban environments: a Meta-analysis. Psychol. Rep. 124, 417–437. doi: 10.1177/0033294119884063, 31694463

[ref33] Mitchell K. S. Dick A. M. DiMartino D. M. Smith B. N. Niles B. Koenen K. C. (2014). A pilot study of a randomized controlled trial of yoga as an intervention for PTSD symptoms in women. J. Trauma. Stress. 27, 121–128. doi: 10.1002/jts.21903 24668767

[ref34] Nordbrandt M. S. Sonne C. Mortensen E. L. Folkersen S. Carlsson J. Vindbjerg E. (2020). Trauma-affected refugees treated with basic body awareness therapy or mixed physical activity as augmentation to treatment as usual-a pragmatic randomised controlled trial. PLoS One 15:e0230300. doi: 10.1371/journal.pone.0230300, 32163509 PMC7067472

[ref35] Noseworthy M. Peddie L. Buckler E. J. Park F. Pham M. Pratt S. . (2017). The effects of outdoor versus indoor exercise on psychological health, physical health, and physical activity behaviour: a systematic review of longitudinal trials. Int. J. Environ. Res. Public Health 20:1669. doi: 10.3390/ijerph20031669, 36767034 PMC9914639

[ref36] Oppizzi L. M. Umberger R. (2018). The effect of physical activity on PTSD. Issues Ment. Health Nurs. 39, 179–187. doi: 10.1080/01612840.2017.1391903, 29319376

[ref38] Qi W. Gevonden M. Shalev A. (2016). Prevention of post-traumatic stress disorder after trauma: current evidence and future directions. Curr. Psychiatry Rep. 18:20. doi: 10.1007/s11920-015-0655-0, 26800995 PMC4723637

[ref39] Reinhardt K. M. Noggle Taylor J. J. Johnston J. Zameer A. Cheema S. Khalsa S. B. S. (2018). Kripalu yoga for military veterans with PTSD: a randomized trial. J. Clin. Psychol. 74, 93–108. doi: 10.1002/jclp.22483, 28524358

[ref40] Resick P. A. Williams L. F. Suvak M. K. Monson C. M. Gradus J. L. (2012). Long-term outcomes of cognitive-behavioral treatments for posttraumatic stress disorder among female rape survivors. J. Consult. Clin. Psychol. 80, 201–210. doi: 10.1037/a0026602, 22182261 PMC3336190

[ref41] Rocha K. K. F. Ribeiro A. M. Rocha K. C. F. Sousa M. B. C. Albuquerque F. S. Ribeiro S. (2012). Improvement in physiological and psychological parameters after 6 months of yoga practice. Conscious. Cogn. 21, 843–850. doi: 10.1016/j.concog.2012.01.014, 22342535

[ref42] Rosenbaum S. Sherrington C. Tiedemann A. (2015a). Exercise augmentation compared with usual care for post-traumatic stress disorder: a randomized controlled trial. Acta Psychiatr. Scand. 131, 350–359. doi: 10.1111/acps.12371, 25443996

[ref43] Rosenbaum S. Vancampfort D. Steele Z. Newby J. Ward P. B. Stubbs B. (2015b). Physical activity in the treatment of post-traumatic stress disorder: a systematic review and meta-analysis. Psychiatry Res. 230, 130–136. doi: 10.1016/j.psychres.2015.10.017, 26500072

[ref45] Sterne J. A. C. Savović J. Page M. J. Elbers R. G. Blencowe N. S. Boutron I. (2019). RoB 2: a revised tool for assessing risk of bias in randomised trials. BMJ 366:l4898. doi: 10.1136/bmj.l4898 31462531

[ref46] Straud C. L. Siev J. Messer S. Zalta A. K. (2019). Examining military population and trauma type as moderators of treatment outcome for first-line psychotherapies for PTSD: a meta-analysis. J. Anxiety Disord. 67:102133. doi: 10.1016/j.janxdis.2019.102133, 31472332 PMC6739153

[ref47] Streeter C. C. Gerbarg P. L. Saper R. B. Ciraulo D. A. Brown R. P. (2012). Effects of yoga on the autonomic nervous system, gamma-aminobutyric-acid, and allostasis in epilepsy, depression, and post-traumatic stress disorder. Med. Hypotheses 78, 571–579. doi: 10.1016/j.mehy.2012.01.021, 22365651

[ref48] Tan L. Strudwick J. Deady M. Collins K. Bryant R. A. Harvey S. B. (2023). Mind-body exercise interventions for prevention of post-traumatic stress disorder in trauma-exposed populations: a systematic review and meta-analysis. BMJ Open 13:e064758. doi: 10.1136/bmjopen-2022-064758, 37438059 PMC10347470

[ref49] van der Kolk B. A. Stone L. West J. Rhodes A. Emerson D. Suvak M. (2014). Yoga as an adjunctive treatment for posttraumatic stress disorder: a randomized controlled trial. J. Clin. Psychiatry 75, e559–e565. doi: 10.4088/JCP.13m08561, 25004196

[ref50] van Westrhenen N. Fritz E. (2014). Creative arts therapy as treatment for child trauma: an overview. Arts Psychother. 41, 527–534. doi: 10.1016/j.aip.2014.10.004

[ref52] Walker E. A. Unutzer J. Rutter C. Gelfand A. Saunders K. VonKorff M. (1999). Costs of health care use by women HMO members with a history of childhood abuse and neglect. Arch. Gen. Psychiatry 56, 609–613. doi: 10.1001/archpsyc.56.7.609, 10401506

[ref53] Warshaw M. G. Fierman E. Pratt L. Hunt M. Yonkers K. A. Massion A. O. (1993). Quality of life and dissociation in anxiety disorder patients with histories of trauma or PTSD. Am. J. Psychiatry 150, 1512–1516. doi: 10.1176/ajp.150.10.1512, 8379556

[ref54] Watkins L. E. Sprang K. R. Rothbaum B. O. (2018). Treating PTSD: a review of evidence-based psychotherapy interventions. Front. Behav. Neurosci. 12:258. doi: 10.3389/fnbeh.2018.00258, 30450043 PMC6224348

[ref55] West J. Otte C. Geher K. Johnson J. Mohr D. C. (2004). Effects of hatha yoga and African dance on perceived stress, affect, and salivary cortisol. Ann. Behav. Med. 28, 114–118. doi: 10.1207/s15324796abm2802-6, 15454358

[ref56] Whitworth J. W. Ciccolo J. T. (2016). Exercise and post-traumatic stress disorder in military veterans: a systematic review. Mil. Med. 181, 953–960. doi: 10.7205/MILMED-D-15-00488, 27612337

[ref57] Whitworth J. W. Nosrat S. SantaBarbara N. J. Ciccolo J. T. (2019a). Feasibility of resistance exercise for posttraumatic stress and anxiety symptoms: a randomized controlled pilot study. J. Trauma. Stress. 32, 977–984. doi: 10.1002/jts.22464, 31743507

[ref58] Whitworth J. W. Nosrat S. SantaBarbara N. J. Ciccolo J. T. (2019b). High intensity resistance training improves sleep quality and anxiety in individuals who screen positive for posttraumatic stress disorder: a randomized controlled feasibility trial. Ment. Health Phys. Act. 16, 43–49. doi: 10.1016/j.mhpa.2019.04.001

[ref60] Wolf S. Seiffer B. Zeibig J. M. Frei A. Studnitz T. Welkerling J. (2024). A transdiagnostic group exercise intervention for mental health outpatients in Germany (ImPuls): results of a pragmatic, multisite, block-randomised, phase 3 controlled trial. Lancet Psychiatry 11, 417–430. doi: 10.1016/S2215-0366(24)00069-5, 38670127

[ref61] Young-McCaughan S. Peterson A. L. Mintz J. Dondanville K. A. Yarvis J. S. Borah A. M. (2022). Testing the role of aerobic exercise in the treatment of posttraumatic stress disorder (PTSD) symptoms in U.S. active duty military personnel: a pilot study. Cogn. Behav. Ther. 51, 309–325. doi: 10.1080/16506073.2021.2001689, 35001842

[ref62] Yuan Z. Peng C. Yang L. Chen H. (2025). Effects of physical activity on patients with posttraumatic stress disorder: a systematic review and meta-analysis of randomized controlled trials. Medicine 104:e41139. doi: 10.1097/MD.0000000000041139, 39833088 PMC11749738

[ref63] Zatzick D. F. Jurkovich G. J. Gentilello L. Wisner D. Rivara F. P. (2002). Posttraumatic stress, problem drinking, and functional outcomes after injury. Arch. Surg. 137, 200–205. doi: 10.1001/archsurg.137.2.200, 11822960

[ref9003] Zhu L. Li L. Li X. Z. Wang L. (2022). Mind-body exercises for PTSD symptoms, depression, and anxiety in patients with PTSD: a systematic review and meta-analysis. Front. Psychol. 12:738211. doi: 10.3389/fpsyg.2021.738211 35153889 PMC8833099

